# Deciphering the laws of social network-transcendent COVID-19 misinformation dynamics and implications for combating misinformation phenomena

**DOI:** 10.1038/s41598-021-89202-7

**Published:** 2021-05-17

**Authors:** Mingxi Cheng, Chenzhong Yin, Shahin Nazarian, Paul Bogdan

**Affiliations:** University of Southern California, Los Angeles, USA

**Keywords:** Complex networks, Applied mathematics

## Abstract

The global rise of COVID-19 health risk has triggered the related misinformation infodemic. We present the first analysis of COVID-19 misinformation networks and determine few of its implications. Firstly, we analyze the spread trends of COVID-19 misinformation and discover that the COVID-19 misinformation statistics are well fitted by a log-normal distribution. Secondly, we form misinformation networks by taking individual misinformation as a node and similarity between misinformation nodes as links, and we decipher the laws of COVID-19 misinformation network evolution: (1) We discover that misinformation evolves to optimize the network information transfer over time with the sacrifice of robustness. (2) We demonstrate the co-existence of fit get richer and rich get richer phenomena in misinformation networks. (3) We show that a misinformation network evolution with node deletion mechanism captures well the public attention shift on social media. Lastly, we present a network science inspired deep learning framework to accurately predict which Twitter posts are likely to become central nodes (i.e., high centrality) in a misinformation network from only one sentence without the need to know the whole network topology. With the network analysis and the central node prediction, we propose that if we correctly suppress certain central nodes in the misinformation network, the information transfer of network would be severely impacted.

## Introduction

With the SARS-CoV-2 pandemic outbreak, COVID-19 related rumors and misinformation infodemic has become a serious problem. The rapid spread of COVID-19 misinformation provokes the social panic, influences political battles^1^, and propagates some dangerous false/fake rumors, e.g., drinking bleach to cure coronavirus^2^, can cost lives. Academic researchers and government authorities are working intensively to fight COVID-19 infodemic by monitoring, identifying, analyzing, and blocking misinformation^3–6^. Commercial giants such as Facebook^7^, Twitter^8^, Google^9^ are also trying to show their efforts in combating misinformation phenomena. Along these lines, a recent mathematical model  illustrates how governments and social media platforms’ efforts can dis-incentivize the spread of fake news by social media users^10^. Previous works^11–13^ analysing misinformation or fake news focusing on misinformation sentences themselves are mainly from natural language processing aspect, i.e., analyze sentiment, veracity, stance, etc. The social feature of misinformation such as how a piece of fake news spreads from one account/website to its vicinity has also been studied from complex network and statistics aspects^14^. Related machine learning problems such as fake news classification and social bot detection are also well-studied^15^. Understanding how (COVID-19) misinformation evolves and spreads by combining both natural language processing techniques and complex network analysis has not been well-studied.

Network science investigated extensively the mathematical characteristics of social (including collaboration and coauthorship^16^), technological (computer, World Wide Web^17^), biological, semantic^18^ and financial networks^19^ and identified various connectivity mechanisms (e.g., linear and nonlinear preferential attachment^20^, node fitness models^21^, weighted multifractal measure models^22,23^). Various examples exist of complex network techniques applied to natural language processing tasks, and the ways of network construction are different in diverse applications. However, few of these are taking care of full sentences and to the best of our knowledge, we are among the first to analyze the time-varying networks. For example, a document can be converted into a complex network where words are represented as nodes and relationships between words, such as semantic^24^, syntactic^25^, and/or co-occurrence^26^ relationships, are represented as edges. Another branch of research considers chunks of document, i.e., sequence of words, as nodes and similarities between sequences as edges^27^. The exercise of complex network in combination with natural language processing is diverse and most of the time, the extracted complex network is time invariant. In contrast, here, we investigate the mathematical characteristics of time-varying COVID-19 related misinformation network representations (we analyze three such network constructions), where the nodes denote the misinformation sentences and the edges capture the sentence-to-sentence similarity. This allows us to decipher the statistical laws that characterize the COVID-19 misinformation phenomenon.

## Results

In this section, we present misinformation characterization, misinformation network evolution analysis, and misinformation central nodes prediction. We first provide an analysis of the COVID-19 misinformation in terms of popularity. We study the COVID-19 misinformation spread trends and discover that the misinformation mean popularity data are, as a group, indistinguishable from an independently and identically drawn sample from a log-normal distribution. We then present three ways of misinformation network construction and their corresponding analysis. We find that newly constructed misinformation graphs evolve and optimize the network information transfer over time. Formulation of misinformation network with node deletion might better describe the rapidly changing reality of misinformation and reveals the need for new complex network models and tools. At last, we present a deep learning-based misinformation network measures predictor that can work in real time to predict network central nodes. With network centrality measures and our deep-learning predictor, we can identify central nodes in misinformation networks with speed and accuracy, therefore we could combat misinformation by removing those important nodes before they become vital.

**Figure 1:**
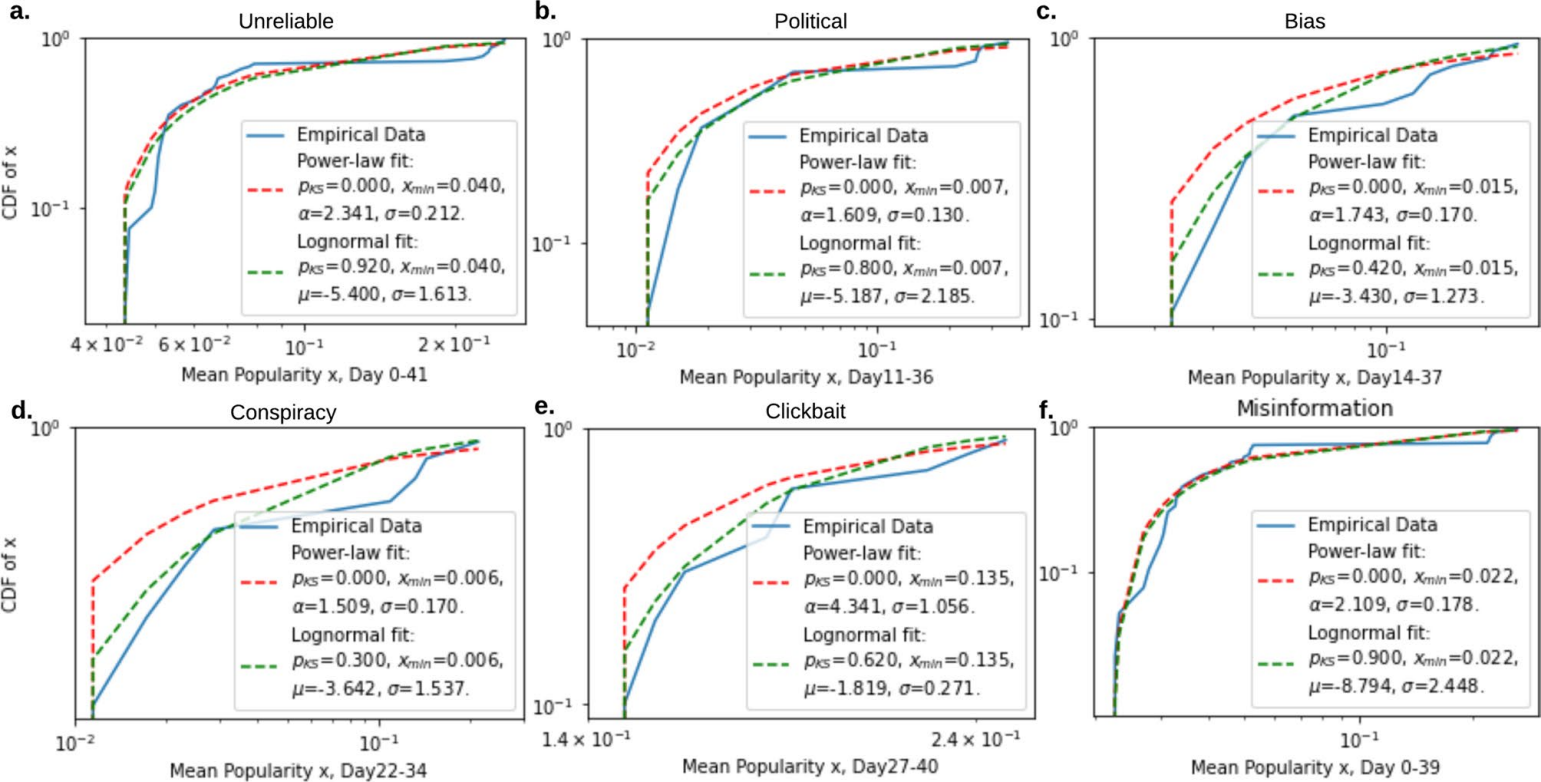
The fitted power-law model (red dash line) and log-normal model (green dash line) of the COVID-19 misinformation mean popularity. (**a–e**) Models fitted for different types of misinformation. (**f**) Models fitted for all COVID-19 misinformation. Log-normal is a plausible data-generating process of the misinformation mean popularity since the plausibility values $p_{KS}$ are greater than 0.1. Both goodness-of-fit test and likelihood ratio test indicate that compared to power-law, log-normal is more plausible. (Detailed hypothesis test procedure is stated in Methods section, “Power-law and log-normal analysis”.) The log-likelihood ratios (*R*’s) and significance values (*p*’s) between the two candidate distributions, log-normal and power-law, are (0.422, 0.429), (0.911, 0.289), (1.832, 0.245), (1.335, 0.352), (1.066, 0.369), (0.565, 0.203), for unreliable, political, bias, conspiracy, clickbait, and all types of misinformation, respectively.

### Characterization

#### Statistical laws characterizing COVID-19 misinformation phenomenon

Researchers have noticed for decades that many measured data retrieved from biological and social sciences can be described by log-normal distribution^28,29^ and power-law distribution^30^. In this work, we estimate the log-normal and power-law models for 5 types of COVID-19 misinformation^31^: unreliable, political, bias, conspiracy, and clickbait misinformation (Data was retrieved from https://usc-melady.github.io/COVID-19-Tweet-Analysis/misinfo.html. Detailed dataset information can be found in Methods section “COVID-19 Misinformation Data”). We use a hypothesis test^32,33^ to estimate model parameters and model plausibility ($p_{KS}$). Estimation methodology can be found in Method section “Power-law and log-normal analysis”. The estimated log-normal model has 3 parameters: $x_{min}$, which represents the smallest value above which the log-normal pattern holds, $\mu$, which is the expected value, and $\sigma$, the standard deviation. Similarly, the estimated power-law model has 3 parameters: $x_{min}$, which represents the smallest value above which the power-law pattern holds, $\alpha$, which indicates the scaling parameter, and $\sigma$, the standard error of the fitted parameter $\alpha$. The parameters of estimated log-normal and power-law models are included in Fig. 1. However, these distribution fitting estimates do not represent that the empirical data, i.e., mean popularity of misinformation in our case, are independent and identically drawn (iid) from the fitted models^30^. We need to evaluate the plausibility of the fitted models quantitatively. Following a standard assessment process, goodness-of-fit test^33^, we find that $p_{KS}$ of log-normal distribution for all 5 types of misinformation and the overall misinformation are much greater than 0.1. That is, log-normal distribution cannot be rejected as a data-generating process.

To further ensure that log-normal rather than power-law distribution is the plausible data generating process, we compare the log-normal distribution and power-law distribution using an appropriately defined likelihood ratio test^32^. The likelihood ratio test provides two values: *R*, the log-likelihood ratio between the two candidate distributions (log-normal and power-law in our case), and *p*, the significance value for the favored direction. If the empirical data is more likely to obey the first distribution, then *R* is positive; otherwise, *R* is negative. The favored distribution is a strong fit if $p>0.05$^32^. As we reported in Fig. 1, log-normal is the favored distribution since the *R* values are all positive and *p* in all likelihood ratio tests are much greater than 0.05. These findings could suggest that the popularity of COVID-19 misinformation could obey a multiplicative process and resembles the generalized Lotka-Volterra (GLV) system^34^. GLV systems are often used to model direct competition and trophic relationships between an arbitrary number of species, e.g., a predator–prey relationship^35^. In this potential misinformation GLV, all kinds of misinformation and individual misinformation generators, e.g., social bots, may be constantly created (and distinguished), and compete with other members in the system for attention.

**Figure 2:**
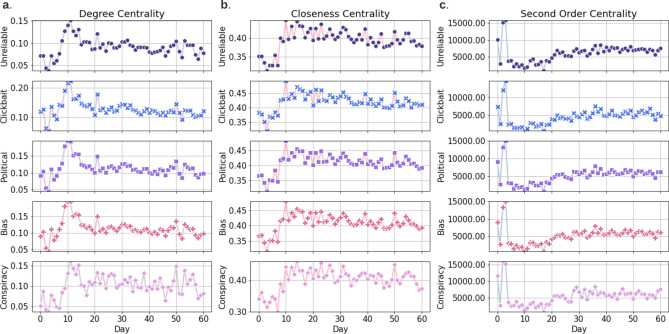
Misinformation network centrality measures. The mean value curves of the degree centrality (**a**), closeness centrality (**b**) and second order centrality (**c**) for misinformation networks of 60 days across five different misinformation categories: unreliable, clickbait, political, bias and conspiracy.

#### Misinformation networks optimize the network information transfer overtime

To characterize misinformation on the semantic level, we construct misinformation networks where nodes and corresponding edges represent the sentences and their sentence similarity, respectively (see Methods section “Misinformation network formulation I” for network formulation details). The new misinformation captured in a day form a distinct misinformation network. In order to investigate the network information transfer characteristics associated with the dynamics of misinformation networks, we quantify their degree-, closeness- and second order-centrality metrics^36,37^. Due to the complex networks’ highly heterogeneous structure, some nodes can be recognized as more significant than others, and centrality measures how important a node is. For instance, in a social network, influencers, recognized as influential nodes with higher centrality, have a lot of followers and can easily propagate specific messages to other nodes. Therefore, calculating the centrality about networks sheds light on information transfer analysis in complex networks^38^.

There are various centrality measures in complex network literature. Degree centrality measures the number of links connected upon a target node and can be utilized to determine the throughput capacity or network localized transmission. The higher the degree centrality is, the higher the chance to receive the information transmitted over a network. Closeness centrality of a node quantifies the average length of the shortest path between the node and all other nodes in a graph and reflects the information transmission latency across a complex network. Thus, the higher the closeness centrality of a node is, the closer it is to other nodes. Second order centrality is a random walk-based betweenness centrality which measures the average time for revisiting a node if following a random walk on a complex network. The standard process of random walk is defined by Newman^39^ where a single node has a probability to direct to a neighbor node (the probability is picked uniformly at random). The higher the second order centrality of a node is, the more information paths pass through it and the less robust the network is to targeted attacks on this node (for details on the degree-, closeness-, and second order-centrality, see Methods section “Networks centrality measures”).

Figure 2a illustrates the mean degree centrality estimated from 60 misinformation networks. Over the first 10 days, the degree centrality of the misinformation networks exhibits an increase tendency towards higher values. It is known that a node achieves an increase in degree centrality by establishing new connections to its neighboring nodes. The high degree centrality of a node means that this node can propagate the received information in an efficient way. Thus, the increasing phenomenon in the first 10 days demonstrates that the misinformation networks tend to optimize their network topology to support higher information flow across the network over time. In addition, when it comes to the last 50 days, the degree centrality enters a relatively stable state which means that after increasing the degree centrality, misinformation networks try to maintain the high speed spread property.

Along the same lines, Fig. 2b shows that the mean of the closeness centrality among 60 misinformation networks across 5 different misinformation categories. In the first 10 days, the mean value of the closeness centrality for misinformation networks is increasing. Higher closeness centrality means that the target node is closer to other nodes and the information sent by the target node can reach other nodes faster. Consequently, this result shows that the misinformation network tends to optimize their network topology to minimize the information transmission latency. In the last 50 days, the mean of the closeness centrality tends to stay stable, which indicates that misinformation networks try to maintain superior transmission latency to keep the network in a high-speed transport state. It is worth noting that the degree- and closeness-centrality are two dual approaches for quantifying information transmission across a network and show a similar network performance optimization behavior in the period of our observation.

Figure 2c shows the second order centrality mean value curves for the 5 misinformation categories in 60 days. On social media, some people periodically delete some old posts. If a post that removed from the network has high second order centrality, the misinformation network has a higher chance to be disconnected. In the first 10 days in Fig. 2c, we observe that the second order centrality exhibits an irregular fluctuation behavior. When it comes to the last 50 days, the second order centrality shows a saturation (slowing in increasing rate) trend, which means that misinformation networks become less-robust/unhealthy over time (a graph is robust/healthy if it is robust to multiple targeted/random attacks^40^). In addition, empirically, a robust graph has most of its elements lying close to each other, and linked by many paths. We conclude that since misinformation networks tend to increase the second order centrality after the early irregular fluctuation, the topology of the misinformation networks becomes more vulnerable to targeted/random attacks over time. In conclusion, the study of the degree-, closeness- and second order-centrality shows that the COVID-19 misinformation networks tend to optimize the information transmission and the topology of the networks becomes more fragile over time.

**Figure 3:**
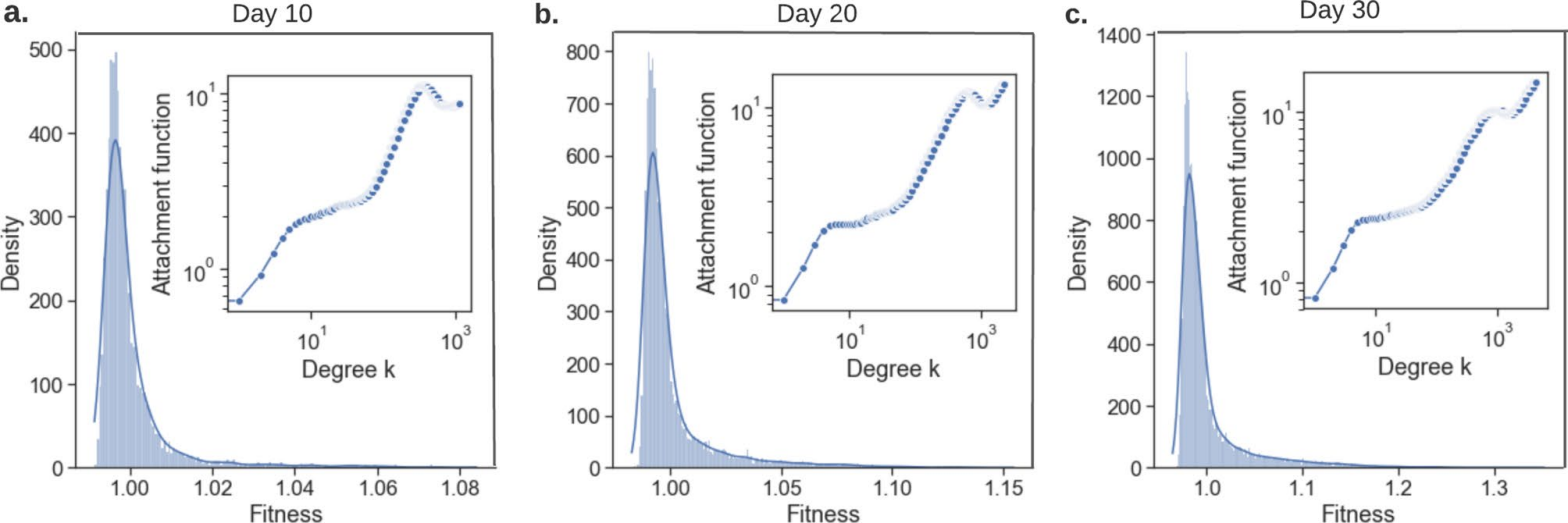
Node fitness and PA function (shown as in-plot) co-estimation for nodes in the misinformation networks on days 10 (**a**), 20 (**b**) and 30 (**c**). The heavy tails of fitness distributions show the existence of the fit get richer phenomenon. The estimated PA functions imply that the higher the node degree, the more competitive the node is in terms of link competition; it also shows a rich get richer phenomenon.

#### Co-existence of fit get richer and rich get richer phenomena in misinformation network

Various mechanisms have been studied to explain the complex network evolution, such as preferential attachment (PA), node fitness theory, node birth/death process. The mapping of network growth onto a Bose-Einstein condensation phenomenon elucidated three phases in the network evolution^41^: a scale-free phase, where all nodes in a network have the same fitness; a fit get richer phase, where nodes with high fitness/quality are more likely to draw new connections; and a Bose-Einstein condensate phase, where the node with largest fitness becomes a clear winner and takes all the new links. In contrast to fit get richer effect, PA is a rich get richer mechanism where nodes with more connections are likely to win more new connections in link competition^42^. The General Temporal model^43^ unifies both PA and node fitness by defining the probability of a node with degree *k* getting new links as $P \propto A_k \times \eta$, where $A_k$ is the PA function and $\eta$ is node fitness (both $A_k$ and $\eta$ are time-invariant).

To show the first evidence of how misinformation network evolves under the assumption of co-existence of PA and node fitness mechanism, we construct the misinformation network by taking the first day’s sentences and construct a base network where nodes are sentences and links represent the sentence similarity. We then grow the network by adding nodes and links as a function of time (days). New misinformation sentences appearing in the next day connect to nodes in the base network if the sentence similarity is over $80\%$. We analyze the PA function $A_k$ and node fitness $\eta$ with PAFit^43^ and the results are shown in Fig. 3 (detailed network growth and analysis methods are described in Methods section “Misinformation network formulation II”). The estimated node fitnesses in day 10, 20 and 30 are all centered around 1, while there exists some nodes with slightly higher node fitness. The heavy-tailed distributions serve as a clear sign of fit get richer effect. From Fig. 3a–c, the maximum node fitness increases, which suggests that fit get richer effect becomes stronger, while the overall effect remains low (the maximum value remains in a medium fitness range [1, 2]). By inspecting the estimated PA function in the in-plots shown in Fig. 3, we make the following two observations: (1) the estimated PA functions $A_k$’s in day 10, 20 and 30 are all increasing with respect to degree *k*, which suggests the existence of a rich get richer effect; and (2) the estimated PA functions are exhibiting a log-linear trend, which matches the widely used log-linear assumption of PA function $A_k=k^\alpha$ as in extended BA model^44^.

#### In misinformation network with node deletion mechanism, node fitness is time-varying and probability of attachment is linear to node degree

**Figure 4:**
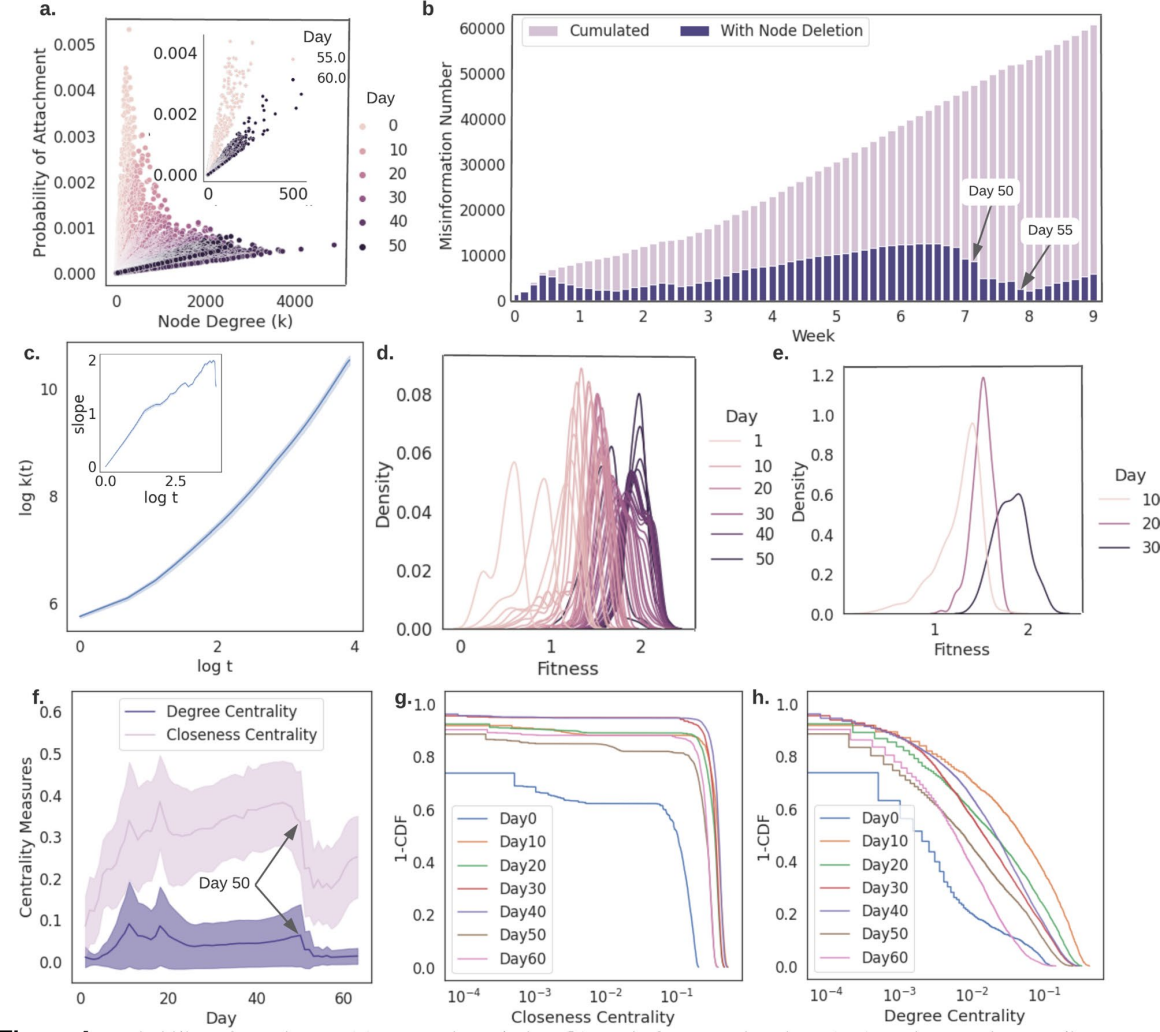
Probability of attachment (**a**), network evolution (**b**), node fitness estimations (**c-e**), and network centrality measures (**f-h**) of misinformation networks with deletion mechanism. (**a**) The probability of attachment is linearly correlated with the node degree. From day 0 to day 50, the probability of attachment for nodes with same degrees is decreasing. This observation shows that the rich get richer effect becomes weaker. Then the probability of attachment from day 55 appears to be similar as in day 0 to 10. (**b**) Misinformation network size (node) evolution comparison. Different from classical network expansion method (as shown in light purple bars), our realistic network construction method with node deletion mechanism appears to be sensitive to public attention/topic shifting. (**c**) The evolution of 158 surviving nodes $S_{[0,50]}$’s accumulated degrees, *k*(*t*) over time. Node fitness is approximated as the slope and it increases from day 0 until day 49 as shown in in-plot. This finding is distinct from the frequently used time-invariant fitness assumption in complex network evolution models. Hence, we further inspect the fitness (growth exponent) distribution of $S_{[0,50]}$ as shown in (**d**). These distributions do not have heavy tail property, which indicates that there is no clear sign of fit get richer phenomenon. Moreover, to compare with fitness distribution we observed in Fig. 3 more clearly, in (**e**), we inspect the fitness distributions of $S_{[0,10]}$, $S_{[0,20]}$, and $S_{[0,30]}$, which correspond to the misinformation network (with node deletion) on day 10, 20, and 30. We find that these non-heavy tail distributions again indicate that there is no clear sign of fit get richer effect. (**f–h**) Network centrality measures. Degree centrality first increases then becomes stable until day 50. This confirms that the rich get richer effect is weak. The closeness is slightly increasing with regard to time and indicates that the newly added nodes lower the average of the shortest path . Nodes with low fitness are deleted in the process, and nodes that survived must maintain high fitness to be close to new nodes. This matches the observations in (**b–c**).

**Figure 5:**
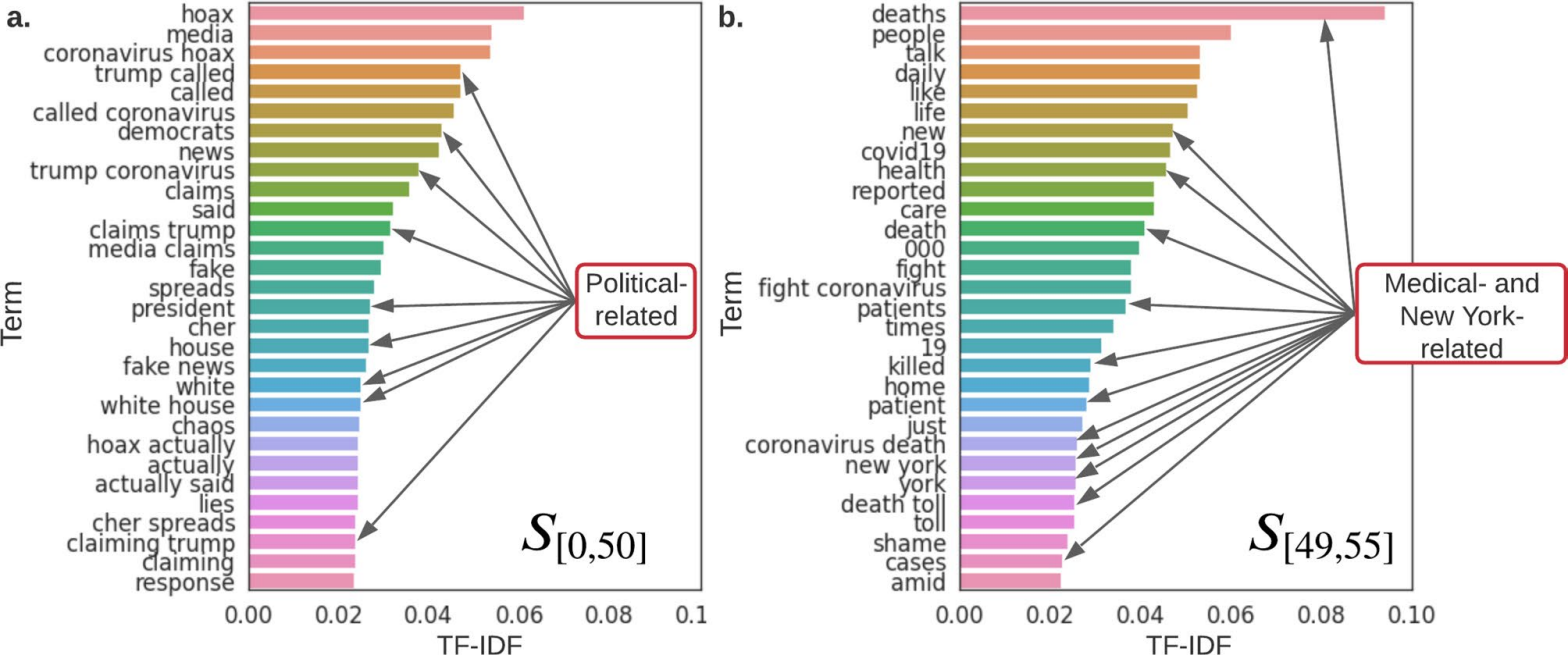
Top words in $S_{[0,50]}$ (**a**) and $S_{[49,55]}$ (**b**). Top words are the words with the highest TF-IDF scores and represent the most influential and important words/topics in sentences. We take n = 1 and 2 for n-grams, therefore, in the results there exist unigrams and bigrams. We find that sentences that survived from day 0 to 50 mainly discussed political-related topics, and sentences that survived from day 49 to 55 are more discussing non-political- or medical-related topics. Specifically, $75.31\%$ in $S_{[0,50]}$, and $41.50\%$ in $S_{[49,55]}$ are discussing political-related topics, respectively. This shift of topic may in fact is the reason for cyclical behavior of probability of attachment we discovered in Fig. 4a.

While the complex network evolution is heavily studied in the literature, the popular models are mostly based on assumptions that PA function and node fitness are time-invariant, and the fundamental network evolution does not consider node deletion mechanism or includes random node deletion mechanism^45^. However, these assumptions are not fully applicable to rapidly changing misinformation networks where people switch attention from one hot topic to another quickly. Under this consideration, we form our misinformation network with a realistic node deletion mechanism, i.e., when a node’s degree is not changing for three days, we delete the node (and its attaching links) from the network with the assumption that this sentence/topic is no longer active or popular at the time. (Detailed network formulation and analysis methods are described in Methods section “Misinformation network formulation III”.) Based on this network formulation method, we estimate the probability of attachment of nodes, node fitness, and network centrality measures and the results are demonstrated in Fig. 4. Firstly, we estimate the probability of attachment of node *j* as $\frac{k_i}{\sum _j k_j}$ as in the BA model^46^, where *k* represents the degree of a node. We find that different from other real-world networks, such as WWW, citation networks, the attachment probability in misinformation networks is linear with respect to node’s degree as shown in Fig. 4a instead of log-linear. This implies that the misinformation network evolution with the consideration of node deletion has weak rich get richer phenomenon. In addition, we observe that the misinformation network evolution experiences expansion-shrink cycles. The slope of the probability of attachment first decreases from day 0 to day 50, then increases to the similar values as in day 0 on day 55. This sudden change between days 50 and 55 shows that the network experiences a destruction and reconstruction phase. We verify this observation by inspecting the network size as shown in Fig. 4b, where the light purple bars represent the cumulative sum of newly emerged misinformation on Twitter (i.e., the afore-mentioned misinformation network constructed in Methods section “Misinformation network formulation I”), and the dark purple bars are the node numbers in the misinformation network constructed with node deletion mechanism. The light purple bars equivalently demonstrate how the misinformation network expands under classical network formulation, which cannot reflect the rapidly changing nature of misinformation network. On the other hand, the dark purple bars demonstrate the network evolution under our realistic misinformation network construction method. It is verified by the dark purple bars that the network does experience a shrink-expand phase between day 50 and 55. In addition, the fluctuations in node centrality measures in Fig. 4f also provide verification. Furthermore, we hypothesize that topic/attention shifting on social media causes this destruction and reconstruction, and we provide evidence in the following discussion and in Fig. 5.

Next, we investigate the node fitness and observe that on day 51, all sentences from day 0 (used for base network) were deleted except one. It is worth noting that we construct our network based on sentence similarity, if some nodes (sentences) in the network do no relate to the newly emerged misinformation, then these nodes are removed from the network. Equivalently speaking, topics or misinformation that are not gaining attention do not fit anymore and will be removed from the misinformation network. If a large-scale node deletion appeared, the misinformation network may experience a destruction phase as we observed previously. Node fitness measures the node quality and reflects the node competitiveness^41^, therefore, we inspect all sentences that survived by day 50 (denoted as $S_{[0,50]}$) and disappear on day 51, and estimate their fitness by tracking the node’s accumulated degree over time *k*(*t*). The slope of *k*(*t*) in a log-log scale, i.e., growth exponent, is therefore equivalent to node fitness^45^ (detailed estimation strategy of node fitness is given in Methods section “Misinformation network formulation III”). Figure 4c–e present the estimated node fitness values and distributions of $S_{[0,50]}$. We find that before a node deletion, its fitness is increasing until two days before deletion. This observation is distinct from the fit get richer phenomenon usually assumed in traditional complex networks without node deletions. When rich get richer and fit get richer are both in play, nodes with high fitness have a higher probability to attract new links and become rich nodes; then, rich nodes reinforce the effect. However, in our network, the rich get richer effect becomes weaker in a cycle, while fitness grows higher. Then, suddenly the nodes with high fitness are deleted at the end of one network evolution cycle. This distinct misinformation network behavior cannot be explained by conventional network models, and may be caused by the rapid attention shift characteristic of social media as we discussed.

We further investigate several hot topics in order to validate the above-mentioned hypothesis on misinformation network evolution. We manually inspect the sentences that survived in the network from day 0 to day 50, noted as $S_{[0,50]}$. Since $S_{[0,50]}$ are all deleted from the network on day 51, and considering our misinformation network construction method, there will be no new links attached to $S_{[0,50]}$. We also study sentences collected on day 49 that managed to survive to day 55, denoted by $S_{[49,55]}$. We compare the *top words*, i.e., the words with highest TF-IDF (term frequency-inverse document frequency^47^) scores, in $S_{[0,50]}$ and $S_{[49,55]}$ as shown in Fig. 5. We find that political words appear the most in the top 30 words of $S_{[0,50]}$ (e.g., “Trump”, “president”, “white house”-related phrases appear about 9 times). In comparison, no political words exist in $S_{[49,55]}$’s top 30 words. This evidence shows that public attention shifts from political-related content to non-political in the time period we investigated. Furthermore, we find that “New York”-related phrases along with medical words such as “deaths”, “killed”, “patients”, “cases” represent the majority of the top 30 words of $S_{[49,55]}$. Which matches the COVID-19 break out in New York from April $18^{th}$ to $24^{th}$. These examples confirm that our network construction method with node deletion mechanism can capture the actual misinformation network evolution. In addition, our network formulation is more sensitive to rapid network changes, e.g., the public attention shift, than classical PA or fitness-based network models.

**Figure 6:**
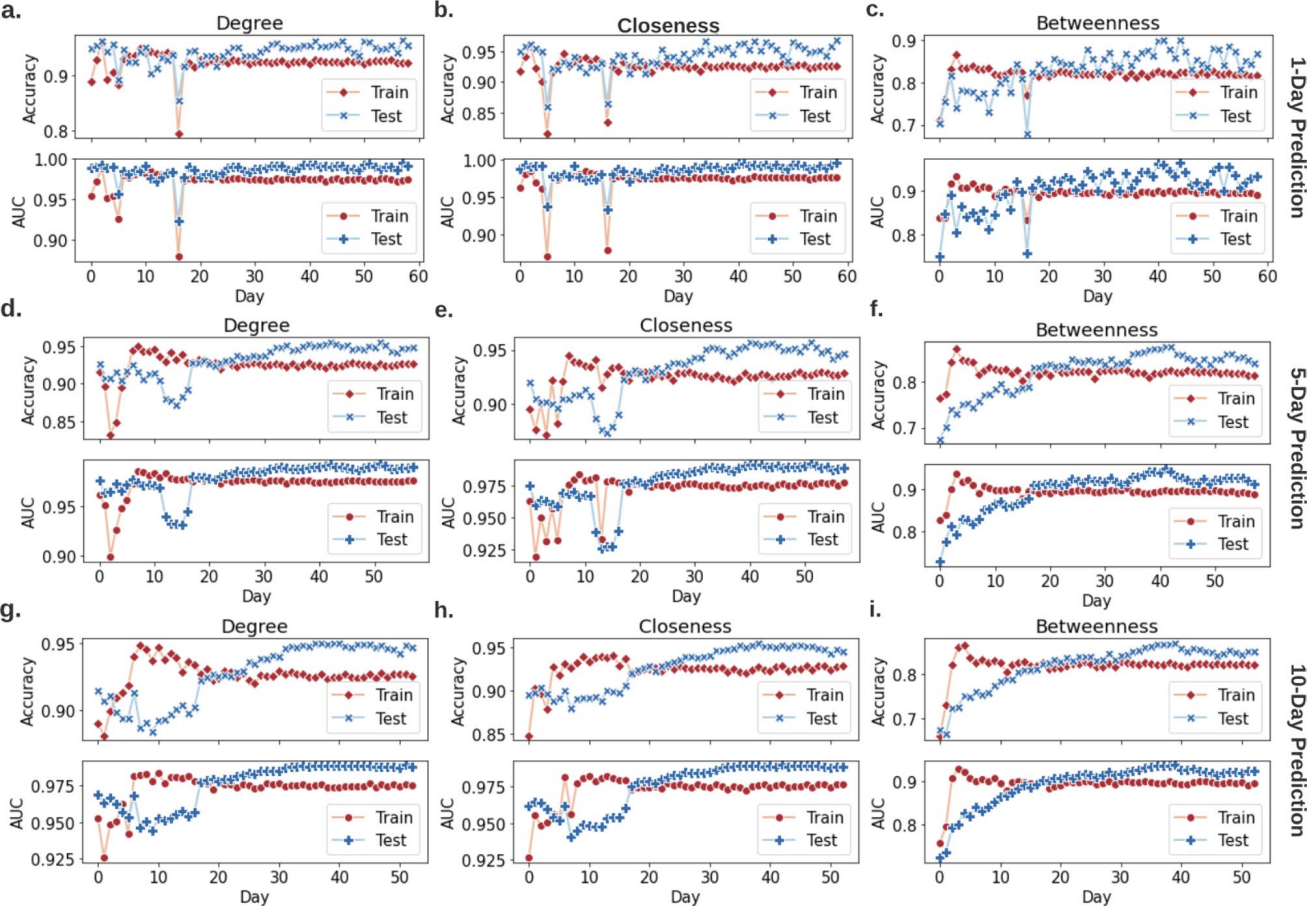
Centrality predictions of daily misinformation networks. To predict day(s) *t*’s central nodes with respect to degree, closeness, or betweenness centrality, daily misinformation networks prior to day(s) *t* are used as training data. Instead of network topology, e.g., adjacency matrix, we take the natural language embedding of each misinformation as the input to the DNN. The DNN then predicts which nodes are going to be the top 100 central nodes in day(s) *t*. E.g., in 1-day prediction, we predict day 10’s top nodes based on day 0–9’s information; and in 5-day prediction, we predict days 5–10’s top nodes based on day 0–5’s information.

### Prediction

#### Deep learning methods accurately predict in real time, which Twitter posts are the central nodes in the misinformation network

Complex network measures such as centrality are calculated based on network topology, i.e., adjacency matrix. However, these metrics are highly computationally expensive and require the adjacency matrix information. In this work, we construct misinformation networks where nodes are sentences, hence, we hypothesize that network measures can be predicted by deep learning and natural language processing (NLP) methods by considering as inputs only the sentences (without adjacency matrix). We verify that complex network metrics of misinformation networks can be easily predicted with high accuracy using deep neural networks (DNNs). In our centrality prediction, to predict day(s) t’s central nodes, we take daily misinformation networks from day t=0 up to day t-1 as training data, and the trained DNN outputs predictions for day(s) t. Specifically, we perform 1-day, 5-day, and 10-day prediction, meaning that for example, in 5-day prediction, if we predict central nodes from day 20 to day 25, we take daily misinformation networks from day 0 to day 19 as training data. In addition, instead of feeding DNN with adjacency matrix, we utilize techniques from natural language processing and feed the DNN with sentence embeddings, specifically, BERT embeddings (training setup can be found in Methods section “Deep learning-based misinformation network measures prediction”). Throughout this process, there is no need to run time-consuming network analysis algorithms, and DNNs predict network measures with high accuracy in real time. Specifically, in 1-day prediction, our DNN predicts degree centrality, closeness centrality, and betweenness centrality, with $94.19 \pm 0.03\%$, $94.25 \pm 0.04 \%$, $83.25 \pm 0.22 \%$ accuracies, and $98.54 \pm 0.01\%$, $98.47 \pm 0.01 \%$, $90.44 \pm 0.21 \%$ AURoCs, respectively, as shown in Fig. 6. The key contributor to this outstanding result is the extracted natural language features in rumors. We believe that the trained neural network learns the syntactic and semantic patterns of influential tweets. This finding enables real time misinformation combat by online identification of fast-spreading and influential misinformation. With an online misinformation detection mechanism, we can utilize the proposed deep learning-based network measure predictor to quickly identify, filter, and delete significant sentences before they actually become the central nodes. Therefore, break the misinformation network before it forms.

## Discussion

Researchers have noticed for a very long time that many measured data retrieved from biological and social systems can be described by log-normal distribution^28,29^, e.g., survival time after cancer diagnosis^48^, number of words per sentence for writers^49^, and size of holes in cocoa cake^29^. During the last decade, power-law distributions are often observed as well, e.g., size of wars^30^. In this work, we analyze the trends of COVID-19 misinformation spread and discover that the log-normal distribution cannot be rejected as a plausible model for misinformation mean popularity data. With COVID-19 credible and unreliable information pushed to smart devices in real time across the globe, the true/false information constantly competes for finite collective attention in this COVID-19 infodemic. The log-normal distribution may suggest that the popularity of COVID-19 misinformation can obey a multiplicative process and resembles to the GLV, where individual misinformation and generators born and die, and compete for attention. These inspirations could contribute to the future analysis of misinformation collective attention and GLV related modeling and control.

To further decipher the laws behind COVID-19 misinformation network evolution, we construct misinformation networks through three different strategies and analyze these networks from information flow and network evolution aspects. We first construct misinformation networks where nodes are misinformation sentences collected within one day, and links represent their sentence similarity. Each network represents the misinformation that appeared on Twitter within one day and the inspection of these networks shows how the COVID-19 misinformation evolves. Analysis of the network centrality measures, i.e., degree centrality, closeness centrality, and second-order centrality, shows that misinformation first learns to optimize information transfer to be more efficient and then maintains the fast-spreading property. Compared to true information, researchers found that misinformation/fake news spreads differently even in early stages^50^. In addition, false news is discovered to be more novel and spread faster than true news^51^. In our work, we showed from the information transfer aspect that misinformation does evolve to be fast-spreading. However, the optimization of information transfer comes with a price, sacrificing the network robustness. In addition, centrality measures reveal the important nodes/influential misinformation in the network, which lay down the foundation of misinformation control. Currently, the estimation of centrality measures is not only time-consuming, but also requires complete information about the topology (e.g., adjacency matrix) of the misinformation networks. Therefore, with sentences as nodes and sentence similarity as links, we propose a deep learning method to predict the centrality measures with the input of sentence only. Utilizing this method, we can predict the next hot topics or central nodes without the need of knowing the whole network topology^52^, which allows us to filter the potential influential misinformation before it actually becomes a center of attention. Researchers have expressed the concern about blocking information on COVID-19 that blocking can in turn fuel the spread of misinformation^53^. This can be true from the perspective of network information flow revealed in this work. If wrong nodes, e.g., certain nodes with low centrality measures, are deleted from the network, the information transfer of the whole network might be enhanced. In contrast, if we correctly remove certain central nodes, then the information transfer of network would be severely impacted.

After inspecting the misinformation evolution in terms of information transfer, we construct the second series of misinformation networks, where we grow the network from a base network. We first form the base network with day 0’s misinformation. Then we add day 1’s misinformation to the base network; and we grow the network with regard to time (days). With the well-established network science methods, PA and node fitness theory, we find the co-existence of fit get richer and rich get richer phenomena. However, this way of network construction may not capture the true nature of the fast-changing feature of misinformation network due to lack of node deletion mechanism. Without node deletion, the time measure is ignored and a hot topic will remain popular regardless of time, and this is in contradiction with the fact that public attention may shift.

To reveal the true nature of the rapidly evolving misinformation network, we propose a third way of misinformation network construction which grows the topology from the base network, while including the node deletion mechanism to reflect that public may forget things. The determination of the node fitness and probability of attachment show distinct evolution behavior that is not fully explainable by fit get richer and rich get richer effects, i.e., some nodes with high fitness do not attract new connections and are deleted from the network. This distinct behavior may be caused by the public attention shift from one hot topic to another. We also find that different from the time-invariant assumptions in node fitness and PA theories, our misinformation network changes rapidly as well as the node fitness and the probability of attachment. These observations reveal the need for new theoretical network models that can characterize and explain the real world fast-evolving networks such as misinformation networks; and also link the collective attention with network science.

Furthermore, rumors are likely to have fluctuations over time^54^. With the node deletion mechanism, we observe evolution cycles of the misinformation network. The size of the misinformation expands and shrinks cyclically. We also find that the misinformation topics that survived in the network are mostly politically motivated. Our study provides a comprehensive data-driven and data science validation and invalidation of the hypotheses enunciated in^55^. Determining in advance potential targets for fake news is an important aspect of misinformation control^56^. We hope by identifying long-lasting, influential, fast-spreading misinformation in the network, we can help fight the COVID-19 and future infodemics by breaking the network before the increasingly popular nodes become influential; and control the misinformation by inserting combating information into the network. Lastly, through three different network formulations, we find limitations of current widely-used network models and researchers should study alternative novel strategies to properly construct networks from observations.

We believe the findings and analysis of this work contribute new knoledge to the current state-of-the-art fake news/rumor/misinformation research and inter-discipline studies of natural language processing and complex networks. In the future, we foresee that our findings and models can also contribute to fruitful technologies that help combat misinformation, identify fake news in early stages, forecast how popular fake news evolves, spreads, and shifts the public opinion during important events. For instance, as we have exemplified with our deep learning framework, these results can be exploited for developing a technology for detecting and forecasting popular opinions that are likely to become dominant or influential in a fast-evolving heterogeneous network. With our network analysis, to make fake news network to destroy itself, we can insert real news in the network at the lowest price and remove significantly influential false news nodes from the network with the highest reward. However, aside from the positives, more problems need solutions, and more questions require answers. In reality, given that we can only partially observe the misinformation or information network, how can we design accurate and efficient algorithms to reconstruct the whole network from partial, scarce, uncertain, and noisy observations? With strategies to monitor accounts and information flow, how to control the network to make users aware of something? How can we control multiple interacting opinion dynamics that are evolving rapidly? In our future work, we will make an effort to tackle these issues, and in particular, misinformation combating problem, study the interaction between true and false information.

## Methods

### COVID-19 misinformation dataset

We analyzed a COVID-19 misinformation dataset containing misinformation collected from Twitter from March 1st to May 3rd^31^. The data was retrieved with Twitter API service (https://developer.twitter.com/en/docs/tweets/filter-realtime/guides/basic-stream-parameters) using keywords related to COVID-19: ‘Covid19’, ‘coronavirus’, ‘corona virus’, ‘2019nCoV’, ‘CoronavirusOutbreak’, ‘coronapocalypse’, from the platform in real time. We used in total 60798 pieces of misinformation identified to build our misinformation networks. There are 6 categories in the retrieved dataset: unreliable, clickbait, satire, bias, political, and conspiracy. More specifically, the unreliable category is defined to include false, questionable, rumorous and unreliable news. Conspiracy category includes conspiracy theories and scientifically dubious news. Clickbait news is misleading, distorted or exaggerated to attract attention. Political and biased news are in support of a particular point of view or political orientation. In addition, satire is based on the consideration that satire has the potential to perpetuate misinformation^57,58^. However, due to the fact that satire category is extremely small (only 29 tweets are labeled as satire), our analysis only focuses on the other five types. We note that in Fig. 1, the last category “misinformation” contains all the misinformation categories including satire.

### Power-law and log-normal analysis

In this section, we describe the methodology of power-law and log-normal fitting of misinformation mean popularity data. The popularity of a misinformation sentence (tweet) is the number of times it appears on Twitter in the time span of dataset, March 1st to May 3rd. The mean popularity is taken across all misinformation records. There are 5 major types of COVID-19 misinformation in the dataset: unreliable, political, bias, conspiracy, and clickbait. We analyze the power-law and log-normal fits with regard to all 5 types individually and as a whole. By using the *powerlaw* Python package^33^, we perform a statistical hypothesis test analysis as follows: (i) we estimate the parameters, e.g., $x_{min}$, $\alpha$, of the power-law model and the log-normal model via *powerlaw*. (ii) We calculate the goodness-of-fit between mean popularity data and the power-law (and log-normal). Specifically, we inspect a plausibility value $p_{KS}$ in goodness-of-fit test. If $p_{KS}$ is greater than 0.1, the power-law (or log-normal) is a plausible hypothesis for the data. (We will describe how to calculate $p_{KS}$ in detail later.) (iii) We compare hypotheses, power-law and log-normal, via a likelihood ratio test provided in *powerlaw*, e.g., $R,p= distribution\_compare ('lognormal', 'powerlaw')$, where *R* is the log-likelihood ratio between the two candidate distributions. If $R>0$, then the data are more likely to follow the first distribution, otherwise the data are more likely to obey the second distribution. *p* is the significance value for that direction. The favored distribution is a strong fit if $p>0.05$.

Now we describe the procedure of goodness-of-fit test and the calculation strategy of $p_{KS}$^32^. Given a dataset and the hypothesized distribution, e.g., power-law, from which the data are drawn, we calculate $p_{KS}$ based on measurement of the “distance” between the distribution of the empirical data and the hypothesized model. This distance *D* is given by *powerlaw* when we fit the data, which is the “Kolmogorov-Smirnov (KS)” statistic. Next, we generate a large number of power-law synthetic data with the estimated parameters and we fit the synthetic data using *powerlaw*. After fitting the synthetic data, we get the distance of synthetic data and the hypothesized power-law model (fitted by the synthetic data), noted as $D_{syn}$. Then we repeat this procedure by generating 50 sets of synthetic data with 50 $D_{syn}$’s. Finally we calculate $p_{KS}$ as the percentage of $D<D_{syn}$.

**Figure 7:**
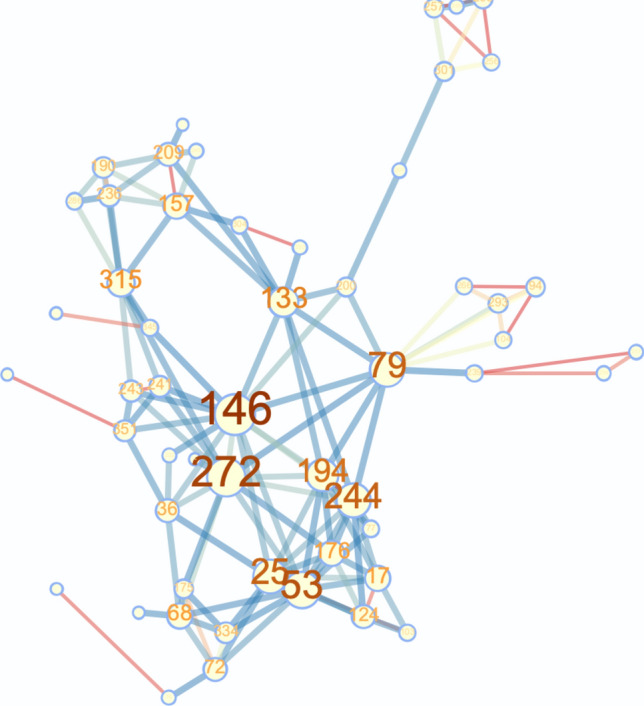
An example misinformation network with 50 nodes (misinformation sentences).

**Table 1:** Top 10 most connected nodes (and their corresponding tweets) in the example network shown in Fig. 7.

Node	Corresponding Misinformation
146	“MOORE, as usual, IS LYING!!Michael Moore: Trump Is Making Coronavirus ‘Partisan’ While Sick People Can’t Get a Test.”
272	“Top Science journal fires off an unusual barrage against Trump for his ‘dangerous’ distortion of coronavirus facts.”
79	“Patton Oswalt Jokes About Trump Supporters Dying of Coronavirus - Big League Politics.”’
53	“Conservative angrily pins blame on ‘Captain Chaos’ Trump for making coronavirus fallout worse than it should be.”
25	“Schumer on Coronavirus: ‘We Are Very Worried About the President’s Incompetence’ This guy is an idiot!”
244	“Congressman erupts on Trump’s health officials for not correcting his ‘bizarre’ lies about coronavirus - Raw Story.”
194	“Congressman erupts on Trump’s health officials for not correcting his ‘bizarre’ lies about coronavirus.”
133	“Donald Trump Jr accuses Hunter Biden of dodging child support as Biden blames coronavirus for skipping court.”
315	“Trying to Cover His Tracks? Trump Reportedly Ordered Coronavirus Talks Classified Common Dreams News.”
157	“‘Stealth attack on Social Security’: Trump condemned for exploiting coronavirus crisis to push tax cut – Raw Story.”

**Figure 8:**
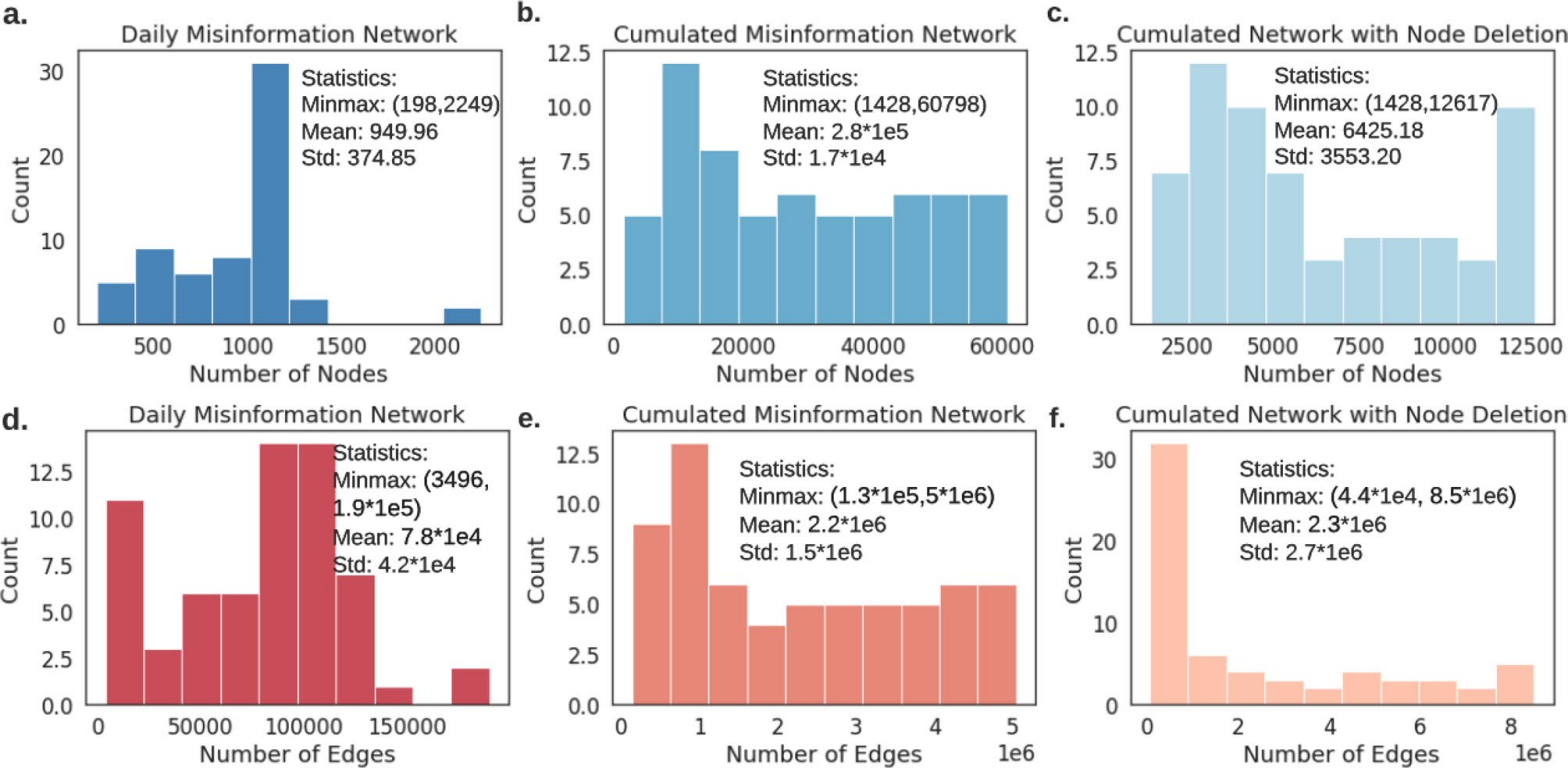
Statistics comparison between networks constructed by formulation I–III. (**a–c**) Node number comparison. (**d–f**) Edge number comparison. We can see that the cumulated misinformation networks (with and without node deletion mechanism) are larger in scale and contain much more nodes and edges compared to daily misinformation networks.

### Misinformation network formulation I

We form networks of new misinformation with respect to time (days). We construct 60 COVID-19 misinformation networks based on misinformation identified on Twitter from March 1st to May 3rd^31^ (days with missing data are discarded). Nodes in a network are sentences, i.e., COVID-19 misinformation, appearing within one day on Twitter. Nodes are connected if two sentences have similarities more than $70\%$. We provide a sample network in Fig. 7, along with original sentences of the top 10 most connected nodes in Table 1. To calculate sentence similarity, we first encode sentences by sentence Transformer model^59^ into vectors with length 786. Then, we measure sentence similarity based on cosine distance. Each misinformation network contains new misinformation that appeared on Twitter per day, and we analyze network features of these networks to characterize how misinformation evolves over time. This distinct choice of network construction comes from the fact that we would like to see how the public opinion and misinformation trends are shifted or evolved from a natural language processing point of view while assuming the emergence of new collective intelligence phenomena. This way of network construction helps us to predict next popular misinformation phenomenon on the social media and helps to combat them.

### Misinformation network formulation II (without node deletion) with PA and node fitness analysis

We construct a misinformation network to capture the evolution of misinformation appeared on Twitter in March 2020. Firstly, we form a base network containing misinformation extracted on March 1st, with the nodes representing misinformation sentences and links indicating the text similarity between two misinformation. We then add nodes and links to the base network based on misinformation extracted from Twitter on the daily basis. Note that we connect the nodes when the text similarity is more than $80\%$ to constrain the network size within a reasonable scale for later analysis. Having the misinformation network, we report the first evidence of co-existence of rich get richer and fit get richer effect in COVID-19 misinformation networks by using PAFit^43^, a general temporal model. To co-analyze both PA and node fitness of a complex network (with the assumption that both fit get richer and rich get richer exist), the probability of a node attracting a new connection is $P \propto A_k \times \eta$, where $A_k$ is the PA function and $\eta$ is node fitness (both are time-invariant). The estimation tasks of $A_k$ and $\eta$ are performed by the R package PAFit.

### Misinformation network formulation III (with node deletion) with the probability of attachment and node fitness analysis

Similarly to the network growth procedure without a node deletion mechanism, we have a base network containing misinformation collected on March $1^{st}$. Then differently than the afore-mentioned monotonic growing process, we include a node deletion mechanism as follows: if a node (sentence) does not attract new connections in $\delta$ consecutive days, we remove this node from the network along with its all edges. The statistics comparison between networks extracted using formulation I-III are shown in Fig. 8. We take $\delta =3$ in this work and links exist only when the text similarity of two nodes is over $80\%$ to keep the reasonable size of the misinformation network. We keep track of this misinformation network from March 1st to May 3rd and estimate the probability of attachment and node fitness. The general temporal model, PAFit, used to measure the misinformation network without node deletion assume the co-existence of fit get richer and fit get richer based on time-invariant PA function and node fitness. However, it may not be applicable to the misinformation network with node deletion. Therefore, we estimate the probability of attachment of each node everyday as $\frac{k_i}{\sum _j k_j}$ using Barabasi-Albert model, where *i* is the target node and *j* represents all other nodes in the network. Node fitness represents how attractive a node is in the network, and it can be estimated as the growth exponent $\beta$^45^. Following Kong et al’s work^45^, assume the cumulative degree of a node *i* at time *t* is *k*(*i*, *t*), and its logarithm reads: $logk(i,t) = (\frac{\eta _i}{A}-\frac{c}{1-c})logt+B = \beta _ilogt+B$, where *A* and *c* are constants, *B* is some time-invariant offset. From this equation, node fitness and the growth exponent are related by a linear transformation, hence the slope of *k*(*i*, *t*) gives an estimation of node fitness value.

### Deep learning-based misinformation network measures prediction

We utilize both deep learning and natural language processing techniques to enable fast network measures prediction. Our DNN takes daily misinformation networks from day 0 to day $t-1$ as training data and predicts which misinformation in day(s) t will end up as central node. The input to our DNN is misinformation sentence embeddings, i.e., BERT embeddings with length 786. The output of our DNN is binary where 0 and 1 indicate a tweet (i.e., a node in a misinformation network) is with low centrality or high centrality, respectively. Our training data is obtained as follows. With 60 misinformation networks, we calculate the centralities via traditional complex network analysis mechanism and take nodes with top 100 centrality measures and label them as 1, otherwise label them as 0. Hence, the training data are misinformation sentences with binary labels. With this way of labeling, the training data end up with imbalanced classes, therefore, we up-sample the minor class to balance the data prior to training. After data balancing, we train a DNN with 3 hidden layers to do binary classification, i.e., to classify if a misinformation sentence is “important” or not. The architecture of our DNN is IN(786)-FC(32)-Dropout(0.5)-FC(32)-Dropout(0.5)-FC(32)-Dropout(0.5)-OUT(2), where IN, FC, Dropout, OUT represent input layer, fully-connected layer, dropout layer, and output layer, respectively, and the number in parenthesis indicates the number of neurons or dropout rate. Fully-connected layers all use ReLU as activation function and output layer uses softmax as activation function. We utilize early stopping training technique to prevent overfitting.

### Network centrality measures

The network centrality measures the importance of a node across a complex network. In this study, the network centralities are calculated by the *NetworkX* package in the Python library^60^. The degree-, closeness-, and second order-centrality are introduced as follows:

Degree centrality^61^ is of node *n* is defined as:

1 $$\begin{aligned} Degree(n)=deg(n) \end{aligned}$$

where *deg*(*n*) is the number of edges connected with the node *n*.

Closeness centrality^62^ of a node measures its average inverse distance to all other nodes and is a way of detecting nodes that can transport information across the network efficiently. The closeness centrality of a node *n* can be defined as follows:

2 $$\begin{aligned} Closeness(n)=\frac{1}{\sum _{u}d(u,n)} \end{aligned}$$

where *d*(*u*, *n*) is the distance between node *u* and node *n*. Of note, $u\ne v$.

Second order centrality^37^ is a kind of random walk based centrality which measures the robustness of the networks. The centrality of a given node *n* is the expectation of the standard deviation of the return times to the node *n* of a perpetual random walk on graph *G*, where the lower that deviation, the more central the node *n* is.
